# Antibodies against AT1 Receptors Are Associated with Vascular Endothelial and Smooth Muscle Function Impairment: Protective Effects of Hydroxysafflor Yellow A

**DOI:** 10.1371/journal.pone.0067020

**Published:** 2013-06-24

**Authors:** Zhu Jin, Wenhui Zhang, Weiran Chai, Yanqian Zheng, Jianming Zhi

**Affiliations:** 1 Department of Physiology, Shanghai Jiaotong University School of Medicine, Shanghai, People’s Republic of China; 2 Department of Assisted Reproductive Technology Shanghai Ninth People’s Hospital Affiliated to Shanghai Jiao Tong University School of Medicine, Shanghai, People’s Republic of China; Max-Delbrück Center for Molecular Medicine (MDC), Germany

## Abstract

Ample evidence has shown that autoantibodies against AT1 receptors (AT1-AA) are closely associated with human cardiovascular disease. The aim of this study was to investigate mechanisms underlying AT1-AA-induced vascular structural and functional impairments in the formation of hypertension, and explore ways for preventive treatment. We used synthetic peptide corresponding to the sequence of the second extracellular loop of the AT1 receptor (165–191) to immunize rats and establish an active immunization model. Part of the model received preventive therapy by losartan (20 mg/kg/day) and hyroxysafflor yellow A (HSYA) (10 mg/kg/day). The result show that systolic blood pressure (SBP) and heart rate (HR) of immunized rats was significantly higher, and closely correlated with the plasma AT1-Ab titer. The systolic response of thoracic aortic was increased, but diastolic effects were attenuated markedly. Histological observation showed that the thoracic aortic endothelium of the immunized rats became thinner or ruptured, inflammatory cell infiltration, medial smooth muscle cell proliferation and migration, the vascular wall became thicker. There was no significant difference in serum antibody titer between losartan and HSYA groups and the immunized group. The vascular structure and function were reversed, and plasma biochemical parameters were also improved significantly in the two treatment groups. These results suggest that AT1-Ab could induce injury to vascular endothelial cells, and proliferation of smooth muscle cells. These changes were involved in the formation of hypertension. Treatment with AT1 receptor antagonists and anti oxidative therapy could block the pathogenic effect of AT1-Ab on vascular endothelial and smooth muscle cells.

## Introduction

Vascular endothelial cells (VEC) are a specifically differentiated tissue. They can release nitric oxide (NO), endothelin (ET), prostaglandin E_2_ (PGE_2_), PGI_2_ and other active substances under normal physiological conditions, participate in material exchange between the blood and the tissues, regulate vascular tension, platelet function, blood coagulation and fibrinolysis, and participate in vascular wall repair [1]. Injury to the endothelial structure and function is therefore believed to be the pathological basis of the development and progression of cardiovascular diseases, tumors and traumatic diseases. Endothelial injury, vascular smooth muscle proliferation, vascular wall thickening and luminal narrowing during the chronic course of hypertension are causes contributing to remodeling changes of the vascular structure.

Angiotensin II (Ang II) is the most important bioactive substance of the renin-angioensin system (RAS), and exerts its physiological actions through AT1 receptors by regulating vascular tension and blood flow, and promoting cell growth and proliferation. Under pathological conditions, over-expression of Ang II in vivo can activate NADPH oxidase (NOX), causing increased expression of intracellular reactive oxygen species (ROS) and pro-inflammatory factors, which not only destroys the intrinsic antioxidant protective mechanism of the blood vessels but reduces NO generation via the NOS melting mechanism, resulting in endothelial dysfunction [2]. In addition, Ang II can also up-regulate the expression of oxidized low-density lipoproteins (ox-LDL) receptor (Lox-1) on the VEC membrane via AT1 receptors (AT1R), leading to VEC dysfunction and promoting the development and progression of atherosclerosis [3], [4]. Ang II can also induce proliferation and hyperplasia of medial smooth muscle cells (SMCs) and cause them to migrate to the intima. As a result, the collagen content is decreased, the contractile ingredients are reduced and the lumen is narrowed. AT1R are the target receptors for Ang II to produce the cardiovascular actions, and selective blockage of AT1R can therefore fully inhibit the RAS. Losatan is a non-peptide specific AT1R antagonist developed in recent years, and plays an increasingly spectacular role in the treatment of cardiovascular diseases.

Studies in recent years have demonstrated that autoimmune response is an important factor in regulating physiological function of the normal cardiovascular system and homeostasis. However, abnormal autoimmune response is a pathogenic factor contributing to and promoting the occurrence of cardiovascular diseases [5]. Since Wallukat el al [6] detected AT1-AA in the serum of preeclamptic patients in 1999, AT1-AA have been detected in the serum of patients with various cardiovascular diseases and those who underwent kidney transplantation [7]. Xia et al reported that AT1-AA were detectable six weeks earlier in the serum of patients with reduced uterine perfusion as compared with the preeclamptic patients. Therefore, AT1-AA is believed to be the important cause for the development of preeclampsia [8]. Further research found that the target point of AT1-AA is in the second extracelluar loop of AT1R (AT1-SEL). It plays an agonist-like effect similar to Ang II, and can increase the beating frequency and the intracellular calcium concentration of neonatal rat cardiomyocytes [6]. It plays an important role in the pathogenesis of cardiovascular diseases by activating NOX [9]. AT1R antagonists and the AFHYESQ peptide segments (181–187 in AT1-SEL) can block the action of AT1-AA. In our previous study used AT1-SEL (165–191) as an antigen to immunize rats, we detected high-titer AT1-Ab, and found that the rat HR was increased and blood pressure as elevated accompanied with structural and functional changes of the heart [10]. However, there is no direct evidence to confirm whether vascular remodeling occurs during the process. For this reason, we established an immunized rat model of the AT1-SEL to see whether or not AT1-Ab participated in the formation of hypertension by changing the vascular structure and function. In addition, we used losartan (an AT1 receptor antagonist) and antioxidant hydroxysafflor yellow A (HSYA) as a preventive treatment, in an attempt to provide references for the clinical treatment of related diseases.

## Animals, Materials and Methods

### Ethics Statement

Male SPF Wistar rats aged 10 weeks and weighing 200–220 g provided by the Experimental Animal Center of Shanghai Jiaotong University School of Medicine (Shanghai, China) were fed with normal rat chow and tap water ad libitum with a 12∶12 h light-dark cycle (lights on at 19∶00 h) at a constant ambient temperature (23±2°C) and humidity (60% ±5%). All experimental protocols were approved by the Experimental Animal Care and Use Committee of Shanghai Jiaotong University School of Medicine.

### Animals and Experimental Procedures

Thirty-two male Wistar rats were equally randomized into four groups: the control group (n = 8), the immunized group (n = 8), the immunized+losartan group (n = 7), and the immunized+HSYA group (n = 8). The animals were actively immunized using artificially synthesized peptide segments corresponding to the sequence of the AT1-SEL once biweekly for 7 cycles, using the method that we described previously [10]. After three episodes of immunization, animals in the treatment groups were administered with losartan or HSYA until the end of immunization. Losartan (20 mg/kg/day, DuPont Merck,Wilmington, USA) and HSYA (10 mg/kg/day, Shanghai Baozhitang Biotech Co., Ltd. China) was dissolved in PBS through gastric perfusion once per day. Systolic arterial pressure (SAP) was measured by tail-cuff method before each immunization with a BP-6 non-invasive rat tail-cuff system (Tai Meng Technology Co., Ltd., Chendhu, China), and serum AT1-Ab titer was measured by tail bleeding method.

### ELISA Detection of Antibodies

Serum antibody titer was measured by using the ELISA method described previously [10]. Briefly, the peptides were coated (10 mg/ml in 100 mM Na2CO3) on 96-well plates. The wells were then saturated with PMT [phosphate-buffered saline supplemented with 5% (w/v) cow sera, 0.1% (V/V) Tween 20, and 0.01% (W/V) Thimerosal (Sigma, St Louis, USA)]. Fifty microliters of serial dilutions (doubling dilutions from 1∶40 to 1∶1280 in PMT) were added to the saturated wells overnight at 48C. An affinity-purified biotinylated goat anti-rat IgG was allowed to react for 1 h, followed by detection using streptavidin– peroxidase (1 mg/ml) (Sigma), and substrates H2O2 (2.5 mM) and 2,29-azino-di (ethylbenzothiazoline) sulfonic acid (2 mM) (Sigma). Optical densities at 405 nm were measured after 30 min by a microplate reader (Molecular Devices, Sunnyvale, USA). P/N ratio <2.1 was set as the negative, and P/N ratio ≥ 2.1 as the positive. P/N ratio = (specimen OD – blank control OD)/(negative control OD – blank control OD). Serum consecutive serial dilution was from 1∶10. The highest dilution at which P/N ≥2.1 appeared was set as the titer of the specimen.

### Determination of Plasma NO, ET and ox-LDL

The rat was anesthetized with pentobarbital sodium (30 mg/g, i.p.), fixed on the operating table and laparotomized. Blood was collected via the abdominal aorta and centrifuged (1000 r/min) at 4°C for 20 min. The serum was kept at −80°C for use. The plasma NO concentration was assayed by nitrate reductase method by reducing NO^−3^ to NO^−2^ according to the reagent assay kit (Jiancheng Bioengineering Research Institute, Nanjing, China). Plasma ET and ox-LDL were assay strictly according to the ELISA kit manufacturers’ instructions (R&D system, US; Mercodia, Switzerland).

### Determination of Vascular Systolic and Diastolic Responses

Immediately after blood collection, the thoracic aorta was removed completely and placed in cold Kreb’s solution to wash out the residual blood. The connective tissue and fat around the aortic segment were cleansed carefully. The same aortic segment about 3 mm was removed from each group for vascular ring tension test (at least four segment per rat aorta), avoiding touching the intima during manipulation for the sake of protecting the integrity of the endothelial cell layer, while the intima of some blood vessels was damaged mechanically. The remaining segment was placed in liquid nitrogen for 4 h, and then transferred to a −70°C freezer for histological test.

Each aortic ring was suspended by means of two parallel stainless steel wires inserted into the lumen in 10 ml jacketed tissue bath containing Kreb’s solution (in mmol/L: NaCl 118,KCl 4.7, KH_2_PO_4_ 1.2, MgSO_4_ 1.2; CaCl_2_ 2.5,NaHCO_3_ 25, Glucose 11.1, EDTA 0.5 mmol) at 37°C and bubbled with 95% O_2_ and 5% CO_2_. One triangle was attached to the bottom of the organ bath and the other was connected to an isometric force transducer (Kent Scientific, Torrington, CT, USA), which was connected to a computerized data acquisition system (PowerLab/8SP, ADInstruments, Castle Hill, NSW, Australia) and recorded on a PC using Chart 5.0 software. Each aortic ring was stretched to a resting tension of 2 g and allowed to equilibrate for 45–60 min, during which the solution was replaced every 15 min.

After equilibration, 60 mmol KCl solution was used to pre-stimulate the blood vessel twice, and then vascular contraction in response to phenylephrine (PE) (10^−8^–10^−4 ^mol/L) was observed. The concentration remained unchanged until the previous contraction was completed. Finally, the PE concentration that made the vascular systolic tension reach the maximum was found out. It was found in the present study that 3×10^−6^ mol/L was the PE concentration that made the vascular systolic tension reach the maximum. The second highest PE concentration (10^−6^ mol/L) used by the pre-contraction vessel was the vascular diastolic function. When the blood vessel reached the PE pre-contraction platform by using the 10^−6^ mol/L concentration, endothelial-dependent diastolic response and non endothelial-dependent diastolic response were observed by using 10^−8^–10^–5^ mol/L acetylcholine (ACh) and 10^−10^–10^−7^ mol/L sodium nitroprusside (SNP). The blood vessel was pre-treated with 10^−4^ mol/L L-NAME and 10^−5^ mol/L indomethacin to observe the effect of NO produced by endothelial nitric oxide synthase (eNOS) and prostaglandin produced by cyclooxygenase (COX) on ACh-induced diastole. The diastolic response was expressed as the percentage of the diastolic force in the PE pre-systolic tension. The maximum systolic/diastolic tension (T_max_/E_max_) and the concentration at which 50% of T_max_/E_max_ (EC_50_/PD_2_) was reached were calculated by Scott ratio method [11].

### Histological Observation and Morphological Measurement of the Aorta

The aortic segments taken from the same location of different groups were fixed in 10% formaldehyde, paraffin embedded, sliced into 4 µm sections, stained with hematoxylin and eosin (HE) and Masson trichrome, and observed for the vascular structure under an optical microscope. The intimal thickness/luminal caliber ratio, and the percentage of the number of intimal smooth muscle cell layers and the intimal collagen were measured by a computer image analysis system. The remaining aortic segments were fixed in 2.5% glutaraldehyde and observed for the ultramicrostructure of the arterial wall under a transmission electron microscope (TEM).

### Statistical Analysis

Antibody titers were expressed as geometric mean ± SD, and the other data were expressed as x ± SD. Pairwise differences were verified by *t* test or *x^2^* test. P<0.05 was considered statistically significant.

## Results

### General Data of the Immunized and Control Groups

There was no significant difference in rat body weight, SAP and HR between the groups before the experiment was started. By the end of the experiment, all animals gained weight, although the body weight of the control group was higher than that of the immunized group (312±8 *vs.* 256±7 g). The body weight of losartan and groups was insignificantly lower than that of the control group (292±7 and 286±8 *vs.* 312±8 g).

**Figure 1:**
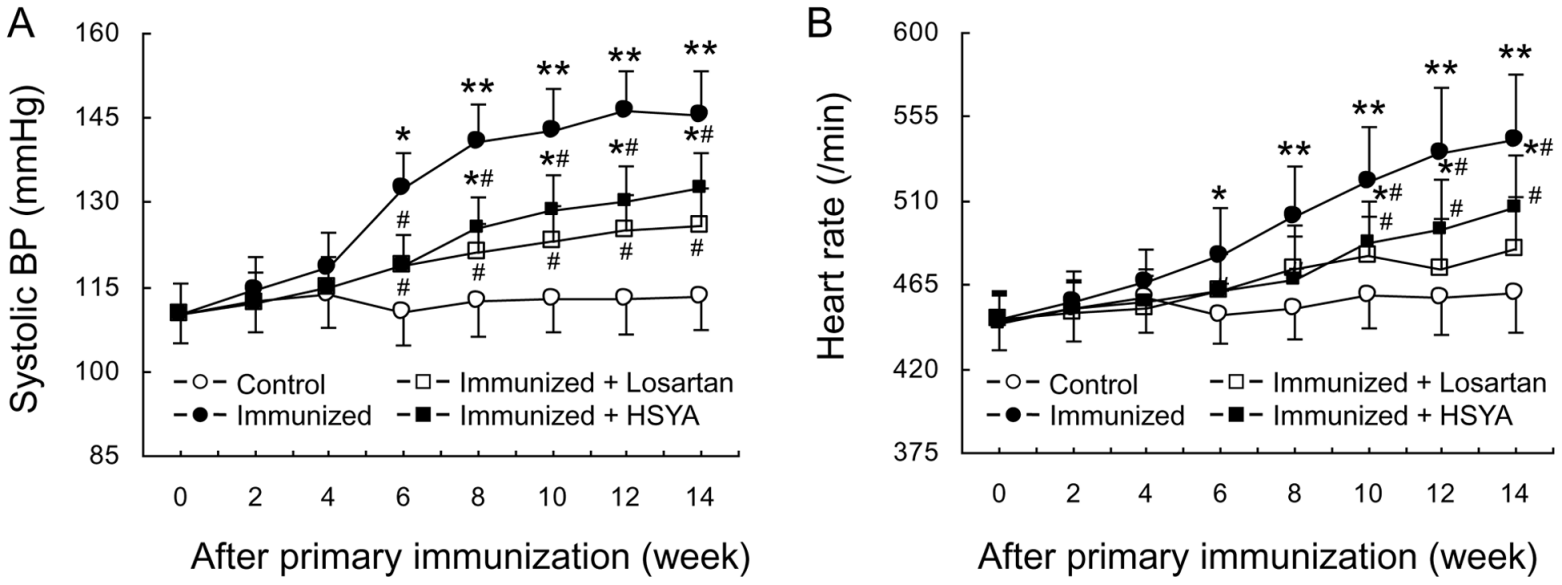
Change in systolic blood pressure (A) and heart rate (B), and antibody titer (C) in different rat groups Data are expressed as the mean ± SD (n≥6). *P<0.05; **P<0.01 *vs*. control group; ^#^p<0.05 *vs*. immunized group.

SAP of the immunized group began rising after three cycles of immunization, and reached a mean of 142.5±7.9 mmHg before the 5^th^ cycle of immunization, and remained at this level thereafter. At the same time, HR was also increased. Both SAP and HR in the treatment groups were lower than those in the immunized group. After several immunizations, SAP and HR of HSYA group were higher than those of the control group, but lower than those of the immunized group. The mean SAP and HR of the control group did not change significantly during the whole course of the experiment (Fig. 1A, 1B).

Serum AT1-Ab did not change significantly in the control group during the whole course of the experiment. AT1-Ab was detectable in the sera of all animals in the immunized group after two cycles of immunization, and the titer reached more than 1∶160 after four cycles of immunization and remained at this level thereafter. The antibody titer reached 1∶2560 in a few animals. AT1-Ab in losartan and HSYA groups changed in a way similar to the immunized group (Fig. 1C).

### Detection of Plasma Biochemical Indexes

**Figure 2:**
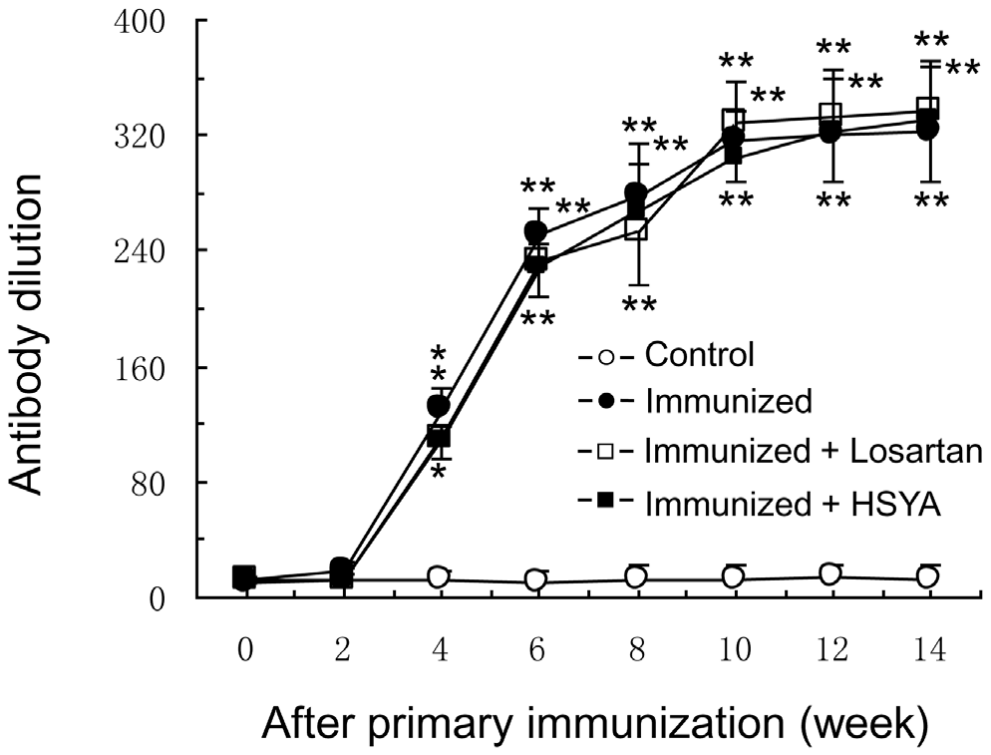
The plasma concentrations of NO, ET and ox-LDL in different rat groups. ET and ox-LDL levels increased significantly and NO level decreased significantly in the immunized group compared with the control group. These parameters were reversed significantly in losartan and HSYA groups. Data are expressed as the mean ± SD (n≥6). *P<0.05; **P<0.01 *vs*. control group; ^#^p<0.05, ^##^p<0.01 *vs.* immunized group.

Compared with the control group, plasma ET and ox-LDL were increased significantly and plasma NO was decreased significantly in the immunized group. The AT1-Ab-induced increase of ET and ox-LDL and decrease of NO were attenuated markedly in losartan and HYSA groups. Compared with the control group, there were some insignificant changes in ET, ox-LDL and NO in losartan and HYSA groups (Fig. 2).

### Aortic Systolic Response

**Figure 3:**
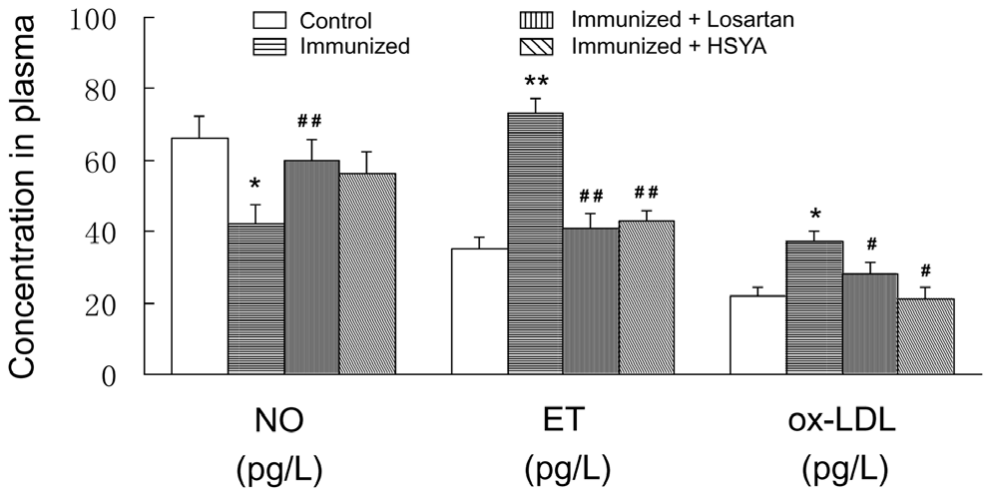
Concentration-response curves of contractile response of the aortic ring with intact endothelium to PE. The concentration-response curves to PE, fitted with Scott equation, in each group are shown (A). Calculated EC_50_ and Tmax of the concentration-response curves to PE are shown (B). Data are expressed as the mean ± SD (n≥6). *P<0.05; **P<0.01 *vs*. control group; ^#^p<0.05 *vs.* immunized group.

The contractile force of the aortic ring treated with10^−6^ mol/L PE was 60∼70% of 60 mm KCl. The contractile force of the aortic ring in response to different concentrations of PE in the immunized group was significantly higher than that in the control group. The dose-response curve moved upward significantly, and the mean maximum contractile force was 1.52 fold that of the control group, the difference being statistically significant. The dose-response curve in response to PE moved downward significantly in losartan and HSYA groups, and the mean maximum contractile force was 1.15 and 1.24 fold that of the control group respectively, the difference being statistically insignificant. EC_50_ in the immunized group was significantly smaller than that in the control group, while EC_50_ in losartan and HSYA groups was not significantly different from that in the control group (Fig. 3).

### Aortic Diastolic Response

#### Diastolic response to ACh and SNP

**Figure 4:**
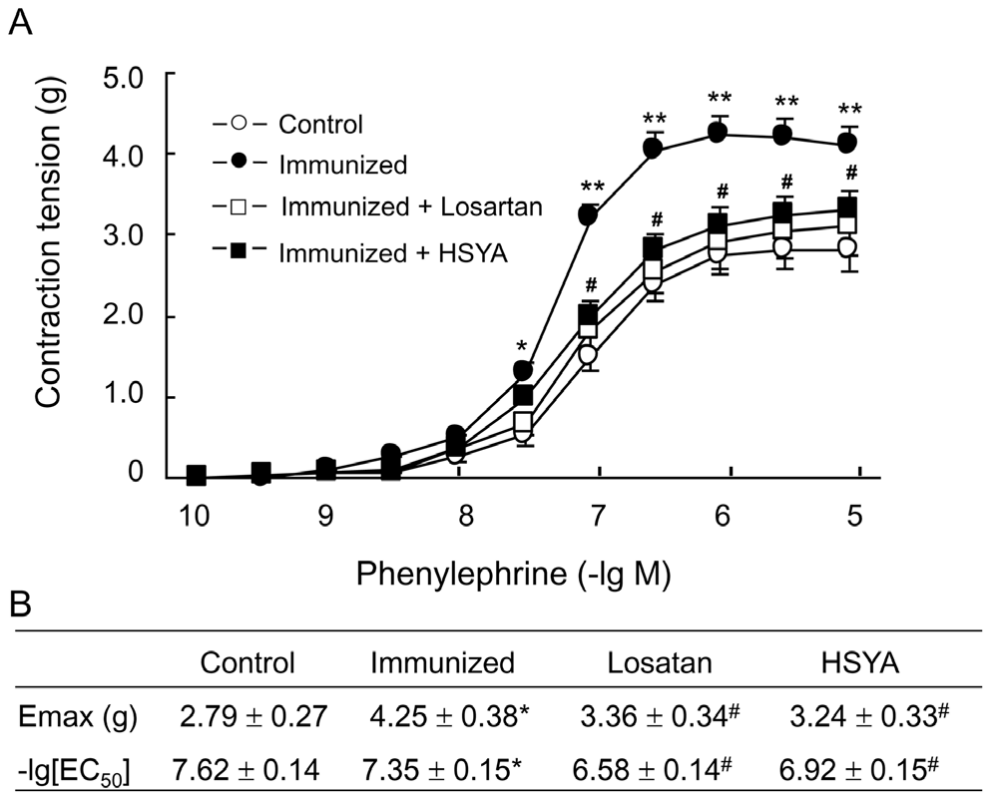
ACh-induced vascular relaxation of the aortic ring with intact endothelium. The aortic ring isolated from the experimental animals was pre-contracted with 10^−6^ M PE, and vascular relaxation in response to ACh was measured. The concentration-response curves to ACh, fitted with Scott equation, in each group are shown (A). Calculated E_max_ and PD_2_ of the concentration-response curves to ACh are shown (B). Data are expressed as the mean ± SD (n≥6). *P<0.05; **P<0.01 *vs*. control group; ^#^p<0.05 *vs.* immunized group.

**Figure 5:**
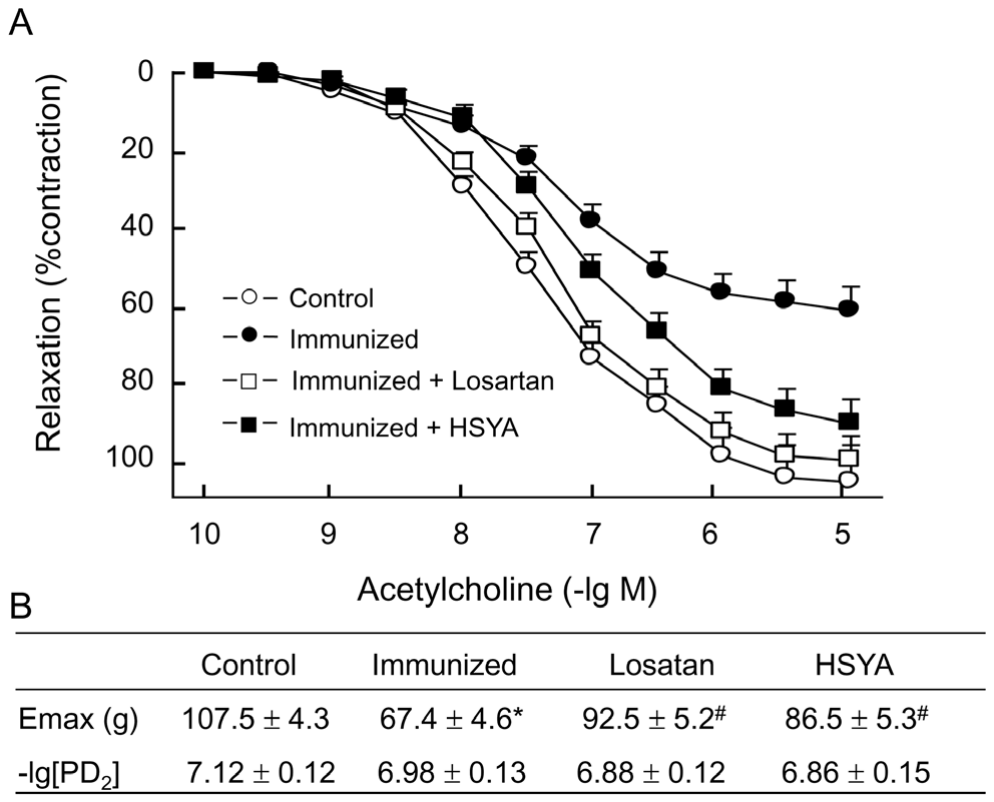
SNP-induced vascular relaxation of the aortic ring with intact endothelium. The aortic ring isolated from the experimental animals was pre-contracted with 10^−6^ M PE, and vascular relaxation in response to SNP was measured. The concentration-response curves to SNP, fitted with Scott equation, in each group are shown (A). Calculated E_max_ and PD_2_ of the concentration-response curves to SNP are shown (B). Data are expressed as the mean ± SD (n≥6). *P<0.05; **P<0.01 *vs*. control group; ^#^p<0.05 *vs.* immunized group.

The aortic ring pre-treated with PE had a dose-dependent diastolic response to ACh, and the maximum diastole was 107.5±4.3%. Compared with the control group, the diastolic curve in response to ACh moved upward in the immunized group, and the maximum response was only 67.4±4.6% (P<0.05 *vs.* control). The diastolic response to ACh recovered substantially in losartan and HSYA groups, and the maximum diastolic response was 92.5±5.2% and 86.5±5.3% respectively. Compared with the immunized group, the diastolic response to ACh in these two groups was increased significantly (P<0.05) (Fig. 4). Observation of the non endothelial-dependent diastolic function in the de-epithelialized blood vessels by using SNP after PE contraction showed that all blood vessels produced dose-dependent diastole, and there was no significant difference in Emax and PD_2_ between the groups (Fig. 5).

### The Effect of L-NAME and Indomethacin on ACh Action

**Figure 6:**
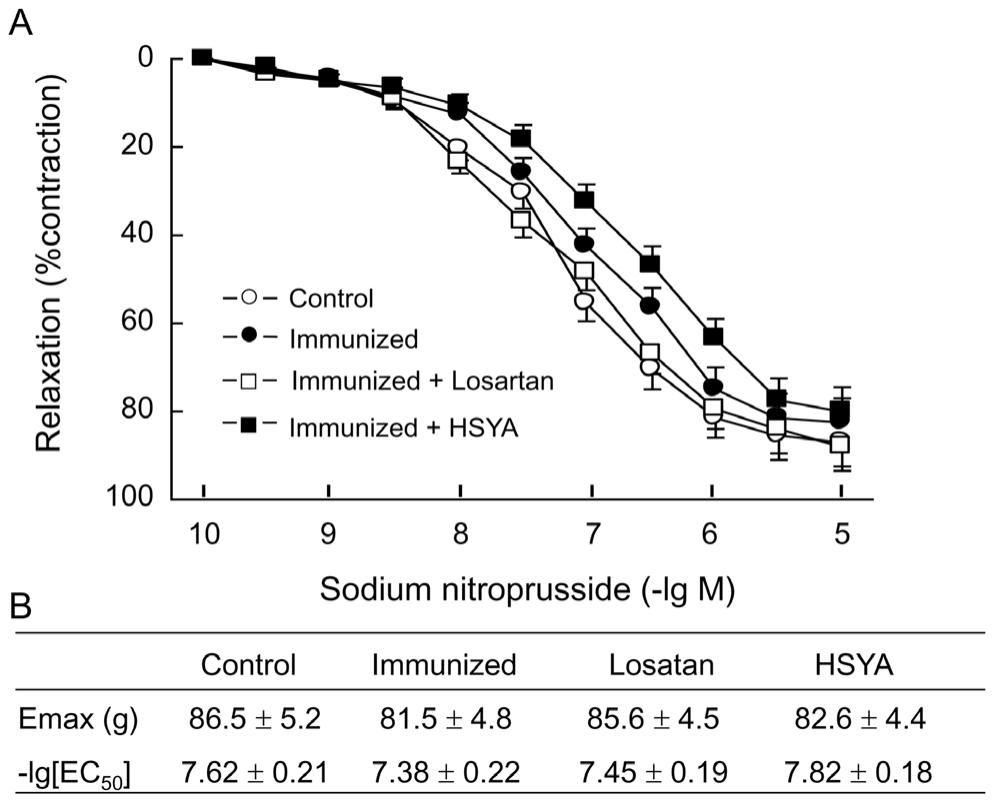
The maximum diastolic value (Emax) of ACh alone and in the presence of 10^−5^ mol/L of indomethacin or 10^−4^ mol/L of L-NAME in aortic ring with intact endothelium. Data are expressed as the mean ± SD (n≥6). *P<0.01 *vs*. ACh alone.

The vascular diastolic action induced by ACh is associated with multiple substances, especially NO and PG. To confirm whether the diastolic function induced by ACh is associated with NO and PG, the vascular effect of ACh was observed after pre-treatment of the aortic ring with NOS inhibitor L-NAME (NN-nitro-L-arginine-methyl ester) and non selective COX inhibitor indomethacin. As is shown in Fig. 6, the maximum value of diastolic response (Emax) induced by ACh was attenuated markedly after pre-treatment of the aortic ring with 10 mol/L L-NAME in all groups (P<0.01), while there was no significant change in Emax after pre-treatment with indomethacin (P>0.05). There was no significant difference in PD_2_ between the groups (data not shown).

### Morphological Observation of the Aorta

**Figure 7:**
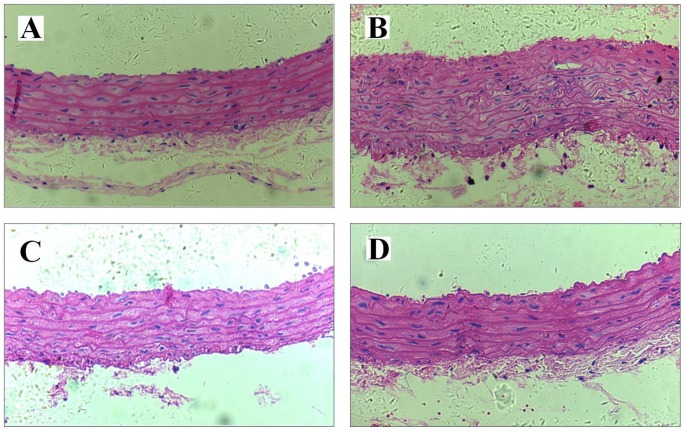
High resolution light microscopy. Representative histological images of the thoracic aorta from control group (A), immunization group (B), immunization+losartan group (C) and immunization+HSYA group (D). There was no significant histological abnormality in control group. In the immunized segment, the aortic intima was thickened, the nuclei of medial smooth muscle cells were densely populated, deeply stained and enlarged; cells were deranged; the number of smooth muscle cell layers between the elastic plates was apparently increased, contrasting with the delicate structure of the intima in control group. Compared with the immunized group, the thickness of the intima and the number of smooth muscle layers in immunized losartan group and HSYA group were decreased; and the medial collagen fibers were arranged regularly. Original magnifications, 200 (A–D).

HE and masson trichrome staining showed that the anatomy of the rat arterial intima, media and adventitia of the control group was clear, with a smooth intima, intact endothelial cells, and little homogeneous wall interstitia component (Table 1). In the immunized group, the aortic intima was thickened in varying degrees, the nuclei of medial smooth muscle cells were densely populated, deeply stained and enlarged; cells were deranged; the number of smooth muscle cell layers between the elastic plates was apparently increased, with more interstitia and disordered arrangement of collagen fibers presenting as larger gaps and disordered layers. In losartan group, the arterial structure was similar to that of the normal group, although there was some proliferation of endothelial and smooth muscle cells. Compared with the control group, the intima of the immunized group was significantly thickened (P<0.01),and the number of smooth muscle layers was also increased. Compared with the immunized group, the thickness of the intima and the number of smooth muscle layers in losartan group were decreased to some extent; the wall interstitial component was also decreased; and the medial collagen fibers were arranged regularly. The structural change of endothelial and smooth muscle cells in HSYA group was similar to that in losartan group (As is shown in Fig. 7).

**Table 1:** Morphometric measurements of aortic arteries from the control group, immunized group, losartan group and HSYA group.

Group	Control	Immunized	Immunized+losartan	Immunized+HSYA
Luminaldiameter (µm)	1548±36	1565±42	1572±43	1593±51
Medialthickness (µm)	137.2±6.2	171.6±8.9*	144.6±7.8#	147.2±7.4#
Media:lumen ratio (×10^−2^)	8.86±0.22	10.96±0.28*	9.19±0.25#	9.24±2.5#
Medial SMC layers	6.28±0.38	11.72±0.48*	6.68±0.38#	7.12±0.42#

* p<0.05 *vs.* control group;

# p<0.05 *vs.* immunized group.

**Figure 8:**
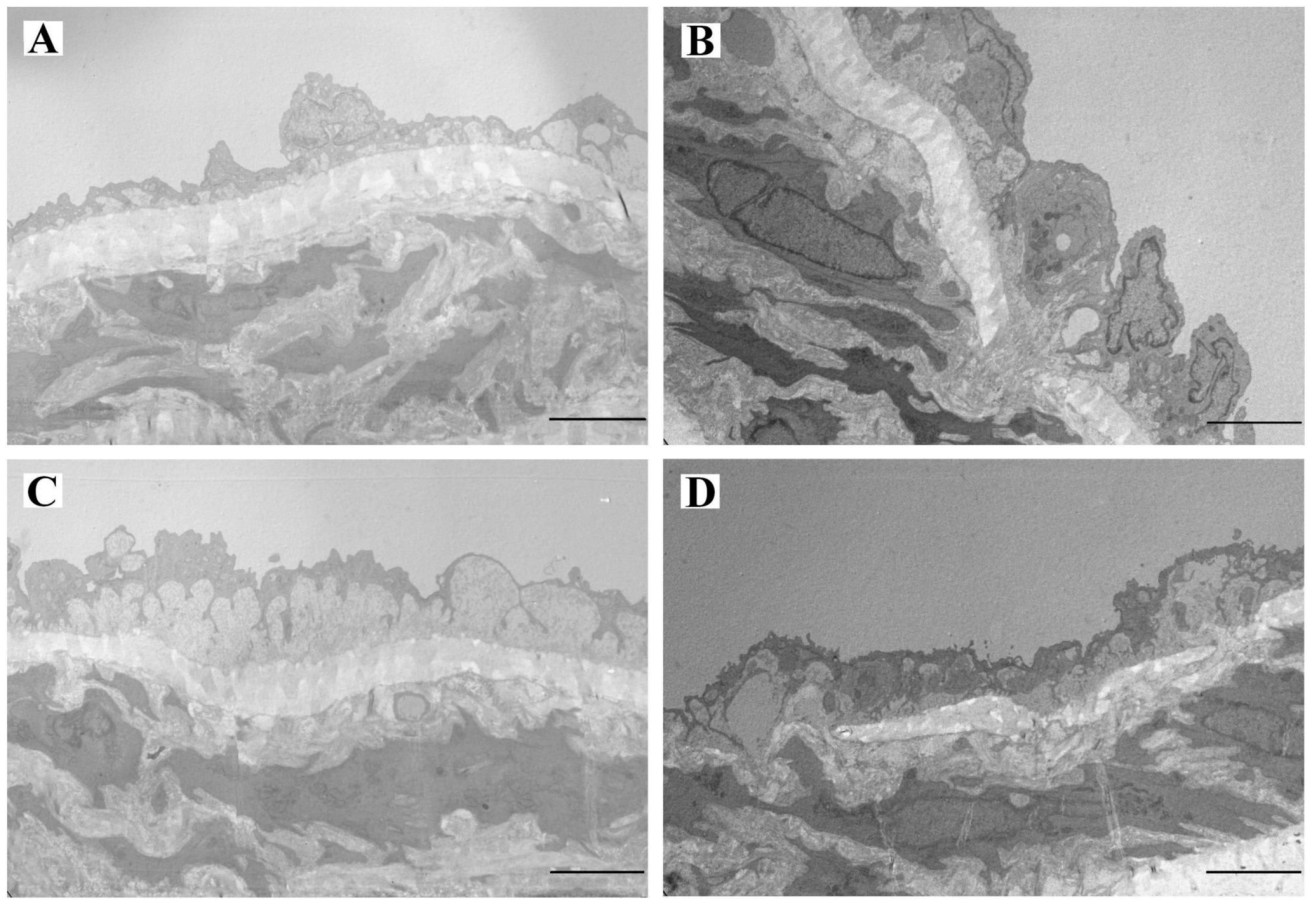
Transmission electron microscopy. Representative views of the thoracic aorta from from different treatment groups. Control group (A), the delicate structure of the intima contains endothelial cells sitting on a basal lamina and internal elastic lamina. Immunization group (B), the intima is thickened. The endothelial cells (end) are heterogeneous, most of them enlarged with convoluted nuclear and cytoplasmic contours. Focal accumulation of mononuclear cells could be detected in the expanded intimal layer. Immunization+losartan group (C) and immunization+HSYA group (D), the ultrastructural changes are qualitatively similar to those found in the immunized rats, although less pronounced, and the tunica intima layer is expanded. The endothelial cells are heterogeneous, most of them enlarged with tortuous nuclear and cytoplasmic contours. Original magnifications 4200.

Optical microscopic observation showed endothelial cells in the normal group were arranged regularly; the internal elastic plates were intact, the medial smooth muscle layer was well differentiated; the cell bodies were fusiform with dense nuclei and contained relatively large amounts of myofilaments. In the immunized group, the endothelial cells were incomplete and deranged; inter-cell connections were widened; and the phenomenon of internal elastic plate rupture was observed. Smooth muscle cells adjacent to the internal elastic plate were enlarged, with abundant organelles and decreased density of myofilaments. Compared with the control group, endothelial changes in losartan and HSYA groups were mild; the internal elastic plates were intact; and there were more organelles, but the content of myofilaments was increased as compared with the immunized group (As is shown in Fig. 8).

## Discussion

Studies in recent years suggest that abnormalities in the autoimmune system are closely associated with the development of cardiovascular diseases. Since Wallukat et al [6] detected AT1-AA in the serum of preeclamptic patients in 1999, AT1-AA have been detected in the serum of patients with various cardiovascular disease. More experimental studies have demonstrated that AT1-AA participate in the development and progression of many cardiovascular diseases. Research on AT1-AA bioactivities has shown that they can act on the AT1-SEL, producing an agonist-like effect similar to Ang II without desensitization [12]. AT1-AA can enhance the effect of Ang II on vascular contraction by altering the spatial structure of AT1R. Wenzel K et al. study reported that they generated and purified activating antibodies against the AT1 receptor (AT1-AB) by immunizing rabbits against the AFHYESQ epitope of the AT1-SEL, then passively transferred AT1-AB into pregnant rats, alone or combined with Ang II. The study results showed that passive transfer of AT1-AB alone or Ang II (435 ng/kg per minute) infused alone did not induce a preeclampsia-like syndrome in pregnant rats. However, the combination (AT1-AB plus Ang II) induced hypertension, proteinuria, intrauterine growth retardation, and arteriolosclerosis in the uteroplacental unit [13]. Zhou CC Et al study found that passive transfer of either total IgG (800 µg) or purified AT1-AA (∼20 µg) from 200 µl donor sera introduced them into either 13-d pregnant mice by retro-orbital injection. Five days later (18 days of gestation), these pregnant mice showed a preeclamptic-like syndrome, including hypertension and proteinuria. The experimental results also found that nonpregnant mice injected with pre-eclamptic IgG that showed a significant increase in blood pressure and proteinuria [14]. AT1R antagonists and 181–187 (AFHYESQ) peptide segments of AT1-SEL could block the effect of AT1-AA [14], [15].

Our previous study showed that blood pressure began rising in rats after induction of AT1-Ab by active immunization. By the end of immunization, the cardiac structure and function had undergone changes, finally forming stable hypertension [10]. In the present study, we further explored the mechanism of AT1-Ab in inducing hypertension, and found that AT1-Ab could cause the aortic endothelium to become thinner or rupture, thicken the vascular wall, and increase the medial thickness/internal diameter ratio as compared with the control group. In addition, the number of medial cell layers was increased; the systolic response of the aorta to PE was increased; and the endothelial-dependent and non endothelial-dependent diastolic responses were decreased markedly. At the same time, the serum endothelin (ET) concentration was increased, while the serum nitric oxide (NO) concentration was decreased. Balance between NO and ET interactions under physiological conditions is an important factor of maintaining the stability of vascular baseline tension. Imbalance between NO and ET is a characteristic feature of arterial endothelial injury. Some studies [16], [17] reported that elevation of the ET concentration induced by AT1-AA is the main cause of hypertension formation. LaMarca et al [16] extracted AT1-AA from the serum of preeclamptic patients and then injected them continuously to pregnant rats via an osmotic pump at an antibody titer of 1∶50, 12 µl daily for 7 consecutive days. It was found that the ET concentration in the renal cortex of the pregnant rats was 11 fold that of the control group. Our study demonstrated that the plasma ET level in the immunized group was significantly higher while the NO level was lower than that in the control group, indicating that the VEC was injured, causing abnormal function.

NOX is a multimeric enzyme existing in many tissues and organs. p47phox is an important subunit of NOX, and its phosphorylation is an initiating step of NOX activity. Knocking out p47phox gene decreased the NOX activity markedly. Dechend et al [8] found that AT1-AA could up-regulate the expression of p47phox subunit of vascular smooth muscle cells and increase NOX activation, producing large amounts of ROS. ROS can oxidize LDL, forming ox-LDL, which has direct toxicity on VEC, causing morphological, structural and functional changes of VEC. In severe cases, it could cause VEC apoptosis and destroy the barrier function of VEC [18]. Some studies [19] reported that activation of AT1-AA could up-regulate the expression of ox-LDA receptor (LoX-1) on the surface of VEC, thus promoting VEC to take up more ox-LDL. The increased amount of ox-LDL in VEC would interfere with the reserve and synthesis of L-arginine (a precursor substance of NO), resulting in insufficient synthesis and release of NO [19]. It was found in this study that ox-LDL increased significantly with the elevation of AT1-Ab in rat serum, probably due to increased NOX activity induced by AT1-Ab and the production of large amounts of ROS, thus increasing the oxidation of LDL. Entry of large amounts of ox-LDL into VEC via LoX-1 receptor would prevent VEC from synthesizing and releasing NO, thus reducing the serum NO level.

The pathological effect of AT1-AA is mainly achieved by activating AT1R. After activation of AT1R, phospholipase C is activated by coupling to Gq/11 and Gi/o to decompose phosphatidylinositol into IP_3_ and DAG, which increases the cytoplasmic Ca^2+^ concentration ([Ca^2+^]i), resulting in Ca^2+^ overload, which in turn induces a series of cellular responses, including promoting VEC to release ET and activating NOX activity of vascular endothelial and smooth muscle cells to produce large amounts of ROS [10]. AT1R antagonists are bioactive substances that block AT1-AA at the receptor level by blocking the NOX activity and reducing ROS production so as to protect the endothelial function and improve vascular remodeling. It was found in our experiment that the use of AT1R antagonists could attenuate the pathological effect of AT1-AB by effectively inhibiting ET release and increasing NO synthesis.

Safflower (Carthamus tinctorius L.) is a traditional Chinese herbal medicine and has the remarkable efficacy of preventing arteriosclerosis and treating coronary heart disease and cerebral infarction. HSYA is a water soluble monomer component extracted from safflower, and its molecule contains several phenolic hydroxyl groups, which are probably related to the antioxidative effect of HSYA. Ample evidence indicates that HSYA can help get rid of free radicals, inhibit lipid peroxidation, and protect the cell membrane. Tian et al [20] reported that HSYA could inhibit ROS release when mitochondrial calcium overload occurs. He et al [21] used HSYA to treat streptozotocin-induced diabetes in a rat model and found that HSYA could inhibit ET generation and ET-induced ROS release. Inspired by these findings, we observed the antihypertensive and anti-oxidative effects of HSYA in the present study. Our results show that HSYA exerted its protective effect on VEC and VSMC by inhibiting AT1-Ab-induced ET release and ox-LDL formation.

The results of the present study show that the plasma ET level was increased markedly in the immunized group; the vascular systolic response of the thoracic aortic ring to PE was increased; and the vascular diastolic response to ACh and SNP was reduced. Medical interventions could increase the reduced diastolic response of the aorta to ACh and SNP in both losartan and HSYA groups, thus attenuating the vascular contractile effect of PE. Although the pharmacological effects of the two drugs are different, both can protect VEC and VSMC against vascular injury induced by AT1-Ab. It is worth to mention that the enhancing effect on vascular diastolic response to ACh and SNP is similar in both losartan and HSYA groups, which is probably due to the fact that the intimal injury and smooth muscle prolifertion and hyperplasia were relatively mild in both groups, indicating that both drugs can be positive to AT1-Ab. However, no preventive therapy has been attempted in asymptomatic patients or patients with mild symptoms. As there was no difference in serum AT1-Ab titer between losartan and HSYA groups and the immunized group, the two drugs are unable to inhibit the process of active immunization.

### Perspectives

This study supports AT1-Ab as a pathogenic factor of preeclampsia and cardiovascular disease. The pathological effect of AT1-Ab is associated with NOX activation and ROS production. Timely treatment with AT1R antagonists and anti-oxidative therapy could prevent cardiovascular disease from occurring in asymptomatic patients or patients with mild symptoms who are positive for AT1-AA.
